# National Trends in Short-term Outcomes Following Non-emergent Surgery for Diverticular Disease

**DOI:** 10.1007/s11605-016-3150-y

**Published:** 2016-04-27

**Authors:** Christina M. Papageorge, Gregory D. Kennedy, Evie H. Carchman

**Affiliations:** Department of Surgery, Division of Colon and Rectal Surgery, University of Wisconsin School of Medicine and Public Health, 600 Highland Ave, K4/730, Madison, WI 53792-7375 USA

**Keywords:** Diverticulitis, Colectomy, Outcomes, Laparoscopy

## Abstract

**Introduction:**

Elective surgery for diverticulitis has evolved over the last decade. We aimed to evaluate the impact of changing practice patterns on postoperative outcomes. We hypothesized that the increased use of laparoscopy, and other management changes, would correlate with a decrease in postoperative complications.

**Methods:**

Patients undergoing non-emergent surgery for diverticulitis from 2005 to 2013 were selected from the National Surgical Quality Improvement Program (NSQIP) database. We compared patient demographics, comorbidities, and operative approach by year of operation using chi-square tests and investigated temporal trends in postoperative outcomes using univariate, trend, and multivariate analyses.

**Results:**

The analytic cohort, which included 29,893 patients, had increasing rates of obesity, advanced age, and higher American Society of Anesthesiologists (ASA) class over the study period. The use of laparoscopy increased significantly from 48 % in 2005/2006 to 70 % in 2013 (*p* < 0.001), while the rate of stoma creation remained unchanged (10–12 %, *p* = 0.072). The absolute risk of any postoperative complication decreased by 5.8 % over the study period, driven primarily by a reduction in infectious complications. Year of operation was a significant independent predictor of fewer complications for 2011–2013.

**Conclusion:**

Despite a trend towards increasing patient complexity, there has been a decline in postoperative morbidity following non-emergent surgery for diverticulitis. This trend coincides with the steadily increasing use of laparoscopy in this population.

## Introduction

Diverticulitis represents a significant disease burden in the United States, with a likely increasing incidence as the population ages and becomes more obese. Diverticular disease accounts for close to 300,000 hospital admissions annually, with an estimated cost of 2.6 billion dollars per year in the USA alone.^1^ Approximately 10–25 % of individuals with diverticulosis will develop diverticulitis, which is the most common indication for colectomy in the USA.^2^,^3^

While colon resection remains the mainstay of elective surgical treatment for diverticulitis, the specifics of surgical management have evolved over the last decade with regard to indications, timing, and type of surgery.^4^–^6^ These changing recommendations and practice patterns have occurred in the setting of improved diagnostic and minimally invasive treatment options as well as an evolving understanding of the natural history and pathophysiology of diverticulitis.^4^ Recurrent episodes of disease were historically thought to occur in approximately 30 % of patients; however, more recent data suggests that the true risk of recurrence is in fact much lower. Prospective data indicates that the risk of recurrence may actually be as low as 1.7 % within 5 years following an episode of acute uncomplicated diverticulitis.^7^ Similarly, younger patients, who were previously thought to suffer from a more virulent form of the disease, have more recently been shown to have comparable outcomes, risk for subsequent attacks, and need for surgery as their older counterparts.^8^–^10^

The most recent practice guidelines published by the American Society of Colon and Rectal Surgeons reflect changing attitudes towards management of diverticular disease. Elective prophylactic partial colectomy for the prevention of future emergency surgery with stoma formation is no longer recommended. These guidelines also de-emphasize the number of attacks and patient age as sole indicators for elective colectomy. In terms of practice patterns, population studies demonstrate a reduction in the proportion of patients undergoing urgent surgery and an increase in the use of percutaneous drainage.^5^,^11^–^14^ Multiple case series endorse the safety of laparoscopic peritoneal lavage for perforated diverticulitis in the acute setting.^15^–^17^ Finally, the use of minimally invasive approaches to colon resection has increased significantly over the last 10–15 years.^5^,^13^

While many authors have examined changes in practice patterns surrounding the surgical management of diverticulitis in the recent decades, little data exists to describe the global impact of these changes on postoperative morbidity. We sought to retrospectively characterize changes in practice patterns and evaluate trends in postoperative outcomes associated with non-emergent surgery for diverticular disease. We hypothesized that the increasing use of laparoscopy and other changes in management will be associated with improved short-term outcomes over time.

## Methods

We performed a retrospective cohort study of a prospectively maintained national clinical database. Patients undergoing non-emergent partial colectomy or colostomy for diverticulitis were divided into cohorts based on year of operation. Baseline demographics, patient comorbidities, operative approach, ostomy usage, and short-term outcomes were then compared across the yearly cohorts. This study was approved by the Institutional Review Board at the University of Wisconsin–Madison.

### Database and Sample Selection

The American College of Surgeons National Surgical Quality Improvement Program (ACS-NSQIP) database was used for this study. This national clinical database, which has been described previously, contains preoperative, operative, and 30-day outcomes data for patients from participating hospitals throughout the country.^18^,^19^ Initially developed in the Veterans Affairs (VA) system as a tool to measure risk-adjusted outcomes, NSQIP began enrolling private sector hospitals in 2004, and data is available from participating hospitals from 2005 onwards. The number of hospitals participating in this database has increased over time from 121 hospitals contributing to the 2005–2006 participant use file (PUF) up to 435 hospitals contributing to the 2013 PUF.^20^

For this study, patients were selected from the 2005–2013 PUFs based on the diagnosis of acute diverticulitis without hemorrhage or diverticulosis without hemorrhage by ICD-9 codes 562.11 and 562.1, respectively. Patients were eligible for inclusion if either the primary procedure Current Procedural Terminology (CPT) code or one of the secondary CPT codes (from the “other procedure” variables) was for partial colectomy or colostomy (Table 1). Exclusion criteria included cases performed emergently, patients of American Society of Anesthesiologists (ASA) class 5 or unknown ASA class, cases performed by a surgical specialist in a field other than general surgery, the presence of preoperative SIRS, sepsis or septic shock, and preoperative ventilator dependence. The patients were then divided into eight cohorts based on year of operation (2005/2006, 2007, 2008, 2009, 2010, 2011, 2012, and 2013).

**Table 1:** CPT codes used for selection of cases for this study

Open	
44140	Colectomy, partial; with anastomosis
44141	Colectomy, partial; with skin level cecostomy or colostomy
44143	Colectomy, partial; with end colostomy and closure of distal segment (Hartmann-type procedure)
44144	Colectomy, partial; with resection, with colostomy or ileostomy and creation of mucus fistula
44145	Colectomy, partial; with coloproctostomy (low pelvic anastomosis)
44146	Colectomy, partial; with coloproctostomy (low pelvic anastomosis), with colostomy
44320	Colostomy or skin level cecostomy
Laparoscopic	
44204	Laparoscopy, surgical; colectomy, partial, with anastomosis
44188	Laparoscopy, surgical, colostomy or skin level cecostomy
44206	Laparoscopy, surgical; colectomy, partial, with end colostomy and closure of distal segment (Hartmann-type procedure)
44207	Laparoscopy, surgical; colectomy, partial, with anastomosis, with coloproctostomy (low pelvic anastomosis)
44208	Laparoscopy, surgical; colectomy, partial, with anastomosis, with coloproctostomy (low pelvic anastomosis) with colostomy

### Endpoints

The operation-related outcomes analyzed in this study included laparoscopic approach and ostomy creation, as defined by the CPT code (Tables 1 and 2). Data regarding conversion from laparoscopic to open approach is not available in this database, and therefore, only cases with a laparoscopic CPT code were categorized as laparoscopic cases. The primary endpoint for comparison of postoperative outcomes across the yearly cohorts was a composite outcome measure of any 30-day postoperative morbidity, which includes occurrence of any of the major 17 complications reported in the NSQIP database. These complications include superficial surgical site infection (SSI), deep SSI, organ space SSI, dehiscence, pneumonia, pulmonary embolism (PE), reintubation, failure to wean from the ventilator, urinary tract infection (UTI), cardiac arrest, cerebrovascular accident (CVA), myocardial infarction (MI), renal insufficiency, renal failure, deep venous thrombosis (DVT), sepsis, and septic shock. Additional composite outcome variables were created, including wound complications (superficial SSI, deep SSI, dehiscence), infectious complications (any type of SSI, pneumonia, UTI, sepsis, septic shock), and non-infectious complications (PE, failure to wean, cardiac arrest, MI, DVT). Finally, occurrence of individual complications, 30-day mortality, and rates of prolonged hospital length of stay (LOS, defined as >7 days) were compared between groups.

**Table 2:** CPT codes used to identify creation of an intestinal stoma

Ostomy creation	
44141	Colectomy, partial; with skin level cecostomy or colostomy
44143	Colectomy, partial; with end colostomy and closure of distal segment (Hartmann-type procedure)
44144	Colectomy, partial; with resection, with colostomy or ileostomy and creation of mucus fistula
44146	Colectomy, partial; with coloproctostomy (low pelvic anastomosis), with colostomy
44320	Colostomy or skin level cecostomy
44188	Laparoscopy, surgical, colostomy or skin level cecostomy
44206	Laparoscopy, surgical; colectomy, partial, with end colostomy and closure of distal segment (Hartmann-type procedure)
44208	Laparoscopy, surgical; colectomy, partial, with anastomosis, with coloproctostomy (low pelvic anastomosis) with colostomy
44310	Ileostomy or jejunostomy, non-tube
44316	Continent ileostomy (Kock procedure)
44322	Colostomy or skin level cecostomy; with multiple biopsies
44187	Laparoscopic ileostomy or jejunostomy, non-tube

### Statistical Analysis

Patient demographics, comorbidities, functional status, and primary diagnosis (diverticulosis versus diverticulitis) were compared by year of operation (2005/2006, 2007, 2008, 2009, 2010, 2011, 2012, and 2013) using chi-square tests. Demographic data included age (categorized as <65 or ≥65 years), gender, and body mass index (categorized as <18.5, 18.5–29.9, or ≥30). All preoperative comorbidities and clinical characteristics reported in the NSQIP database consistently over the course of the study period were analyzed. These include presence of diabetes, chronic obstructive pulmonary disease (COPD), dyspnea on exertion, history of smoking within 1 year prior to surgery, ascites, newly diagnosed or newly symptomatic congestive heart failure (CHF) within 30 days prior to surgery, hypertension, acute renal failure, dialysis dependence, open wound or wound infection, disseminated cancer, chronic steroid use, weight loss of >10 % of body weight in the 6 months prior to surgery, and bleeding disorder or chronic anticoagulation. The “wound infection” variable is defined as any open wound, including, but not limited to, open surgical wounds and enterocutaneous fistulae.

Temporal trends in operative approach (laparoscopic versus open), ostomy creation rates, and postoperative morbidity and mortality were investigated across yearly cohorts in a univariate fashion using chi-square tests. Univariate trend analysis was performed using Mantel-Haenszel tests. Multivariate models using multiple logistic regression to control for differences in patient demographics, comorbidities, primary diagnosis, and operative approach were developed to assess the independent contribution of operative year as a predictor of postoperative outcomes. The year 2005/2006 was considered the reference exposure in this model. Preoperative blood transfusion was excluded as a risk-adjusting variable given the significant definition change in this variable that occurred in 2010. Finally, to further explore the relationship between laparoscopy and postoperative complications, patients were divided into two groups based on operative approach (laparoscopic versus open), and chi-square and Mantel-Haenszel tests were performed to evaluate for trends in postoperative outcomes over time stratified by an operative approach. For all tests, *p* < 0.05 was considered statistically significant.

Missing data was quite minimal in the analytic cohort. There were 60 cases (0.2 %) with missing gender information, 199 cases (0.7 %) with missing BMI data, and 39 cases (0.1 %) with missing functional status. Subjects with missing data were excluded from univariate analyses involving these variables and from all multivariate models.

## Results

**Fig. 1:**
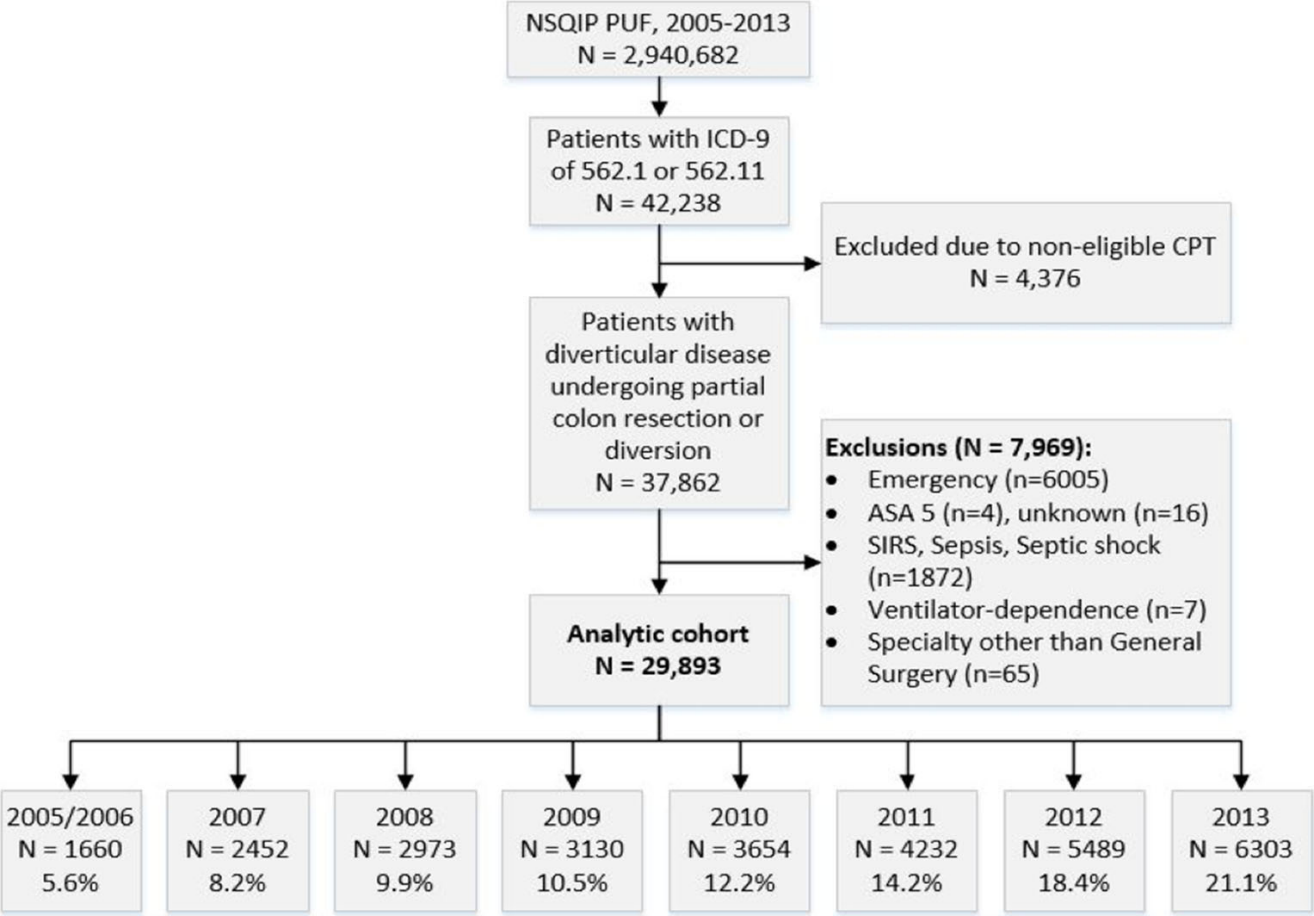
Flow diagram showing patient selection for inclusion in the study and creation of cohorts

From 2005 to 2013, a total of 42,238 patients were identified for potential inclusion in the study based on primary diagnosis (Fig. 1). Of these patients, only 37,862 met inclusion criteria based on CPT procedure codes for partial colon resection or colostomy. An additional 7969 patients met exclusion criteria, yielding a final analytic cohort of 29,893 patients undergoing non-emergent surgery for diverticular disease. The yearly cohorts ranged in size from 1660 patients in the 2005/2006 group to 6303 patients in the 2013 group (Fig. 1).

**Fig. 2:**
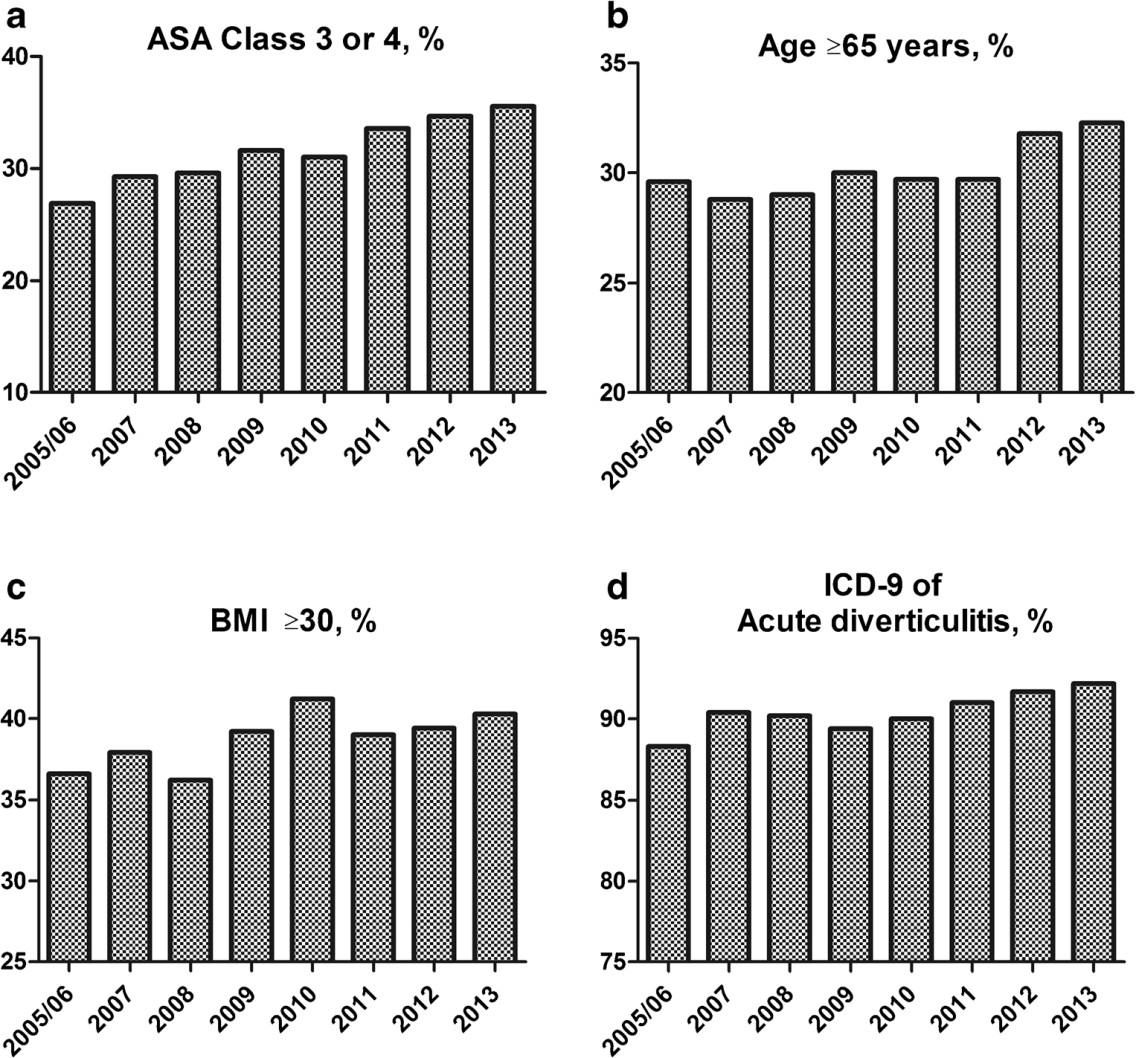
Baseline patient characteristics of each yearly cohort. **a** Percentage of patients who are ASA class 3 or 4 in each yearly cohort (chi-square, *p* < 0.001; Mantel-Haenszel, *p* < 0.001). **b** Percentage of patients aged 65+ years in each cohort (chi-square, *p* = 0.001; Mantel-Haenszel, *p* < 0.001). **c** Percentage of patients with BMI ≥ 30 in each cohort (chi-square, *p* < 0.001; Mantel-Haenszel, *p* < 0.001). **d** Percentage of patients with ICD-9 diagnosis of acute diverticulitis without hemorrhage (rather than diverticulosis without hemorrhage) in each yearly cohort (chi-square, *p* < 0.001; Mantel-Haenszel, *p* < 0.001)

Univariate analysis comparing patient demographics and preoperative comorbidities between the eight cohorts demonstrated significant differences between the groups in gender distribution; age; BMI; presence of diabetes, dyspnea, and ascites; preoperative steroid use; preoperative weight loss of >10 % of body weight; functional status; and ASA class (Table 3). In general, there were temporal trends towards higher ASA class and an increasing percentage of patients with obesity from 2005 to 2013 (Fig. 2a, c). The proportion of patients under the age of 50 years decreased significantly from 30 to 24 % (*p* < 0.001), with a simultaneous increase in the proportion of individuals aged 65 years or older from 29 to 32 % (*p* = 0.001) (Fig. 2b). The proportion of patients classified with the ICD-9 diagnostic code for acute diverticulitis without hemorrhage (562.11) has increased over time with a corresponding decrease in the use of the ICD-9 562.1 (diverticulosis without hemorrhage) (Fig. 2d). The incidence of diabetes has increased over time, while dyspnea, preoperative weight loss, and dependent functional status have all decreased significantly (Table 3). To attempt to further quantify if the overall health of the cohorts has improved or worsened over time, the percentage of patients with two or more comorbidities was calculated for each group and was found to have remained unchanged over time at 23–25 % (*p* = 0.601).

**Table 3:** Baseline characteristics and comorbidities of patients in each yearly cohort

Characteristic^a^	2005/2006, % (*n* = 1660)	2007, % (*n* = 2452)	2008, % (*n* = 2973)	2009, % (*n* = 3130)	2010, % (*n* = 3654)	2011, % (*n* = 4232)	2012, % (*n* = 5489)	2013, % (*n* = 6303)	*χ* ^2^ *p* value
Gender									
Female	52.0	52.4	53.2	55.0	55.4	54.8	53.8	55.5	*0.033*
Male	48.0	47.6	46.8	45.0	44.6	45.2	46.2	44.5	
Age									
<50 years	29.7	28.5	27.9	27.3	25.9	25.9	24.5	24.2	*<0.001*
65+ years	29.6	28.8	29.0	30.0	29.7	29.7	31.8	32.3	*0.001*
BMI 30+	36.6	37.9	36.2	39.2	41.2	39.0	39.4	40.3	*0.011*
Diabetes	8.3	8.4	8.5	9.5	8.9	10.0	9.5	10.1	*0.041*
Smoker	21.0	23.1	21.9	23.1	21.5	21.0	21.1	21.0	0.126
Dyspnea	7.5	6.4	7.3	6.4	6.2	5.2	5.1	4.4	*<0.001*
COPD	3.4	3.6	3.1	3.4	3.5	3.5	3.6	3.2	0.884
Ascites	0.4	0.5	0.2	0.2	0.1	0.1	0.1	0.1	*<0.001*
CHF	0.4	0.2	0.1	0.2	0.2	0.2	0.2	0.3	0.781
Hypertension	45.2	45.1	45.0	45.5	45.9	45.9	45.2	45.4	0.993
Acute renal failure	0.1	0.1	0.03	0.2	0.1	0.05	0.1	0.05	n/a
Dialysis	0.3	0.3	0.3	0.7	0.2	0.3	0.4	0.3	0.064
Disseminated cancer	0.4	0.1	0.2	0.5	0.5	0.4	0.2	0.4	0.100
Wound infection	0.8	1.0	1.1	1.2	1.1	1.1	1.4	1.3	0.447
Steroids	3.7	2.7	2.5	3.2	2.5	3.0	3.4	3.4	*0.029*
Preoperative weight loss	3.5	3.4	2.8	2.5	2.0	2.2	2.2	2.3	*0.001*
Bleeding disorder	1.9	2.6	2.6	2.6	2.4	2.2	2.2	2.0	0.400
≥2 comorbidities	24.6	23.7	23.2	24.6	23.7	23.9	22.9	23.1	0.601
ASA class 3 or 4	26.9	29.3	29.6	31.6	31.0	33.6	34.7	35.6	*<0.001*
Dependent functional status	2.3	1.7	1.8	2.1	1.5	1.2	1.3	0.8	*<0.001*

Italics indicates *p*<0.05

^a^Patients with the following missing data were excluded from this analysis: gender (*n* = 60, 0.2 %), BMI (*n* = 199, 0.7 %), and functional status (*n* = 39, 0.1 %)

**Fig. 3:**
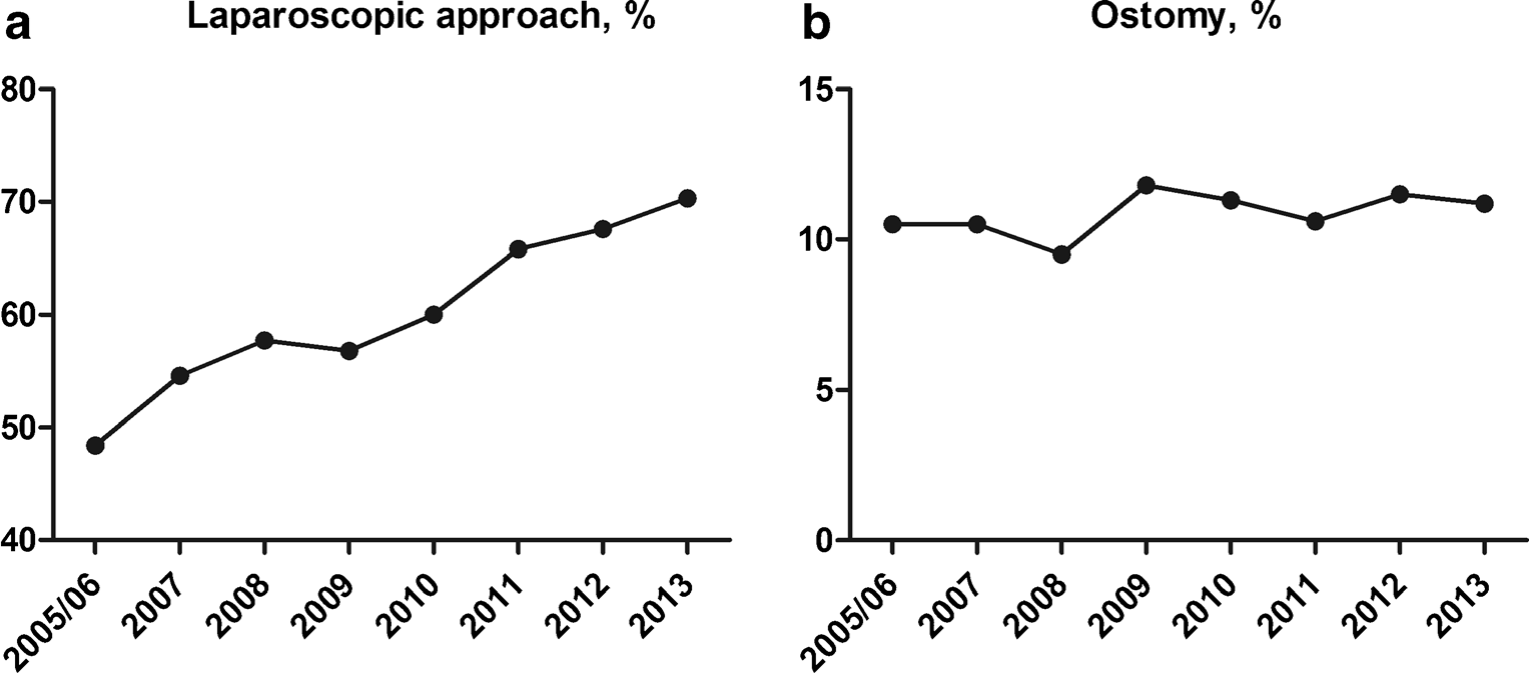
Operative approach characteristics across yearly cohorts. **a** Percentage of patients undergoing laparoscopic approach (chi-square, *p* < 0.001; Mantel-Haenszel, *p* < 0.001). **b** Percentage of patients receiving an ostomy (either ileostomy or colostomy, diverting or end) in each yearly cohort (chi-square, *p* = 0.072; Mantel-Haenszel, *p* = 0.058)

With regard to the operative approach, there has been a dramatic, steady increase in the use of laparoscopy for the surgical management of diverticular disease (Fig. 3a). In the 2005/2006 cohort, only 48.4 % of patients underwent a laparoscopic operation. This percentage gradually increased over time, up to 70.3 % in 2013 (*p* < 0.001). In contrast, rate of ostomy creation, based on CPT codes, has remained relatively constant at approximately 10–12 % over this 9-year period (*p* = 0.072) (Fig. 3b).

**Fig. 4:**
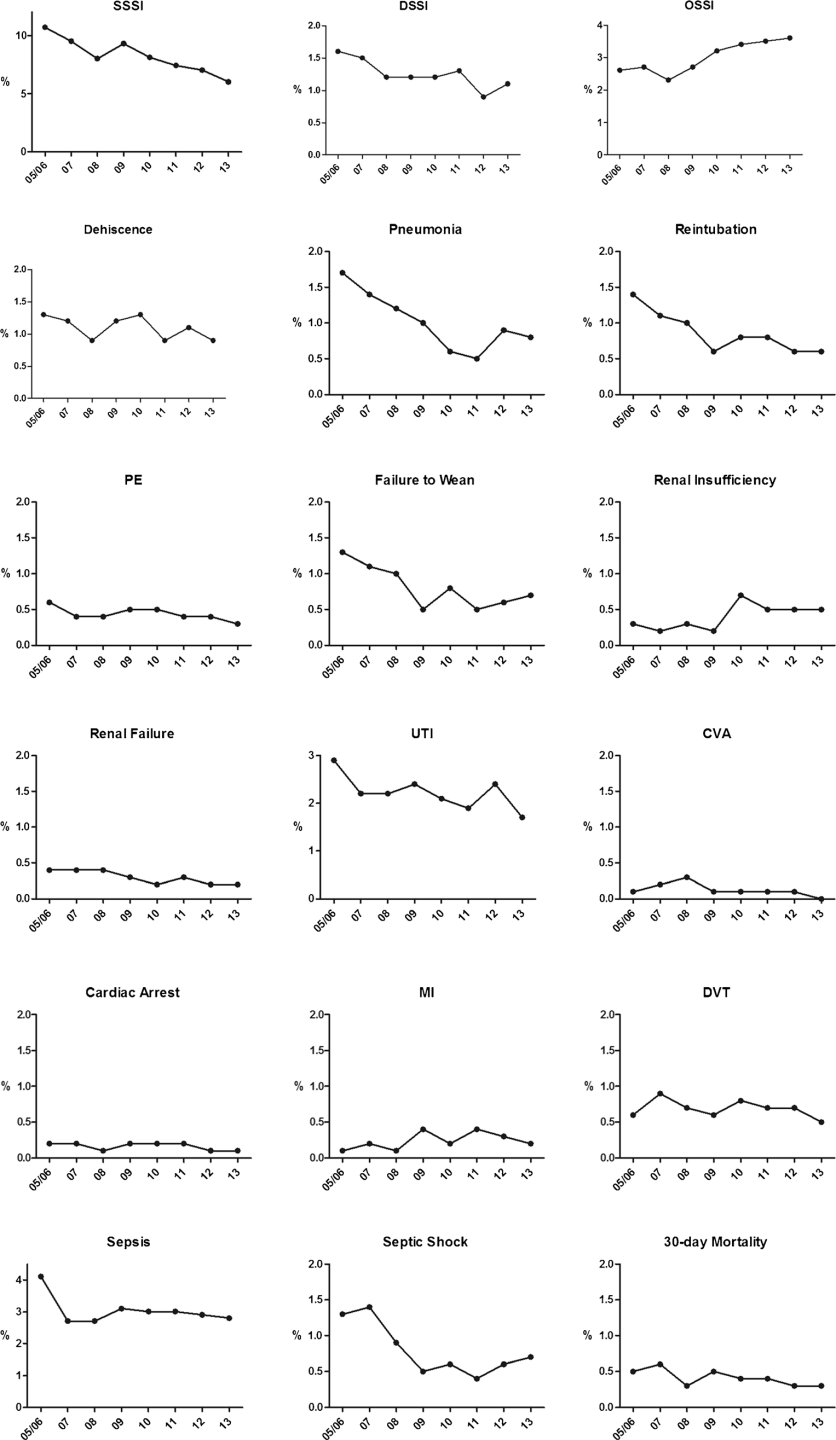
Univariate analysis of 30-day outcomes following surgery for diverticular disease across yearly cohorts from 2005 to 2013

**Fig. 5:**
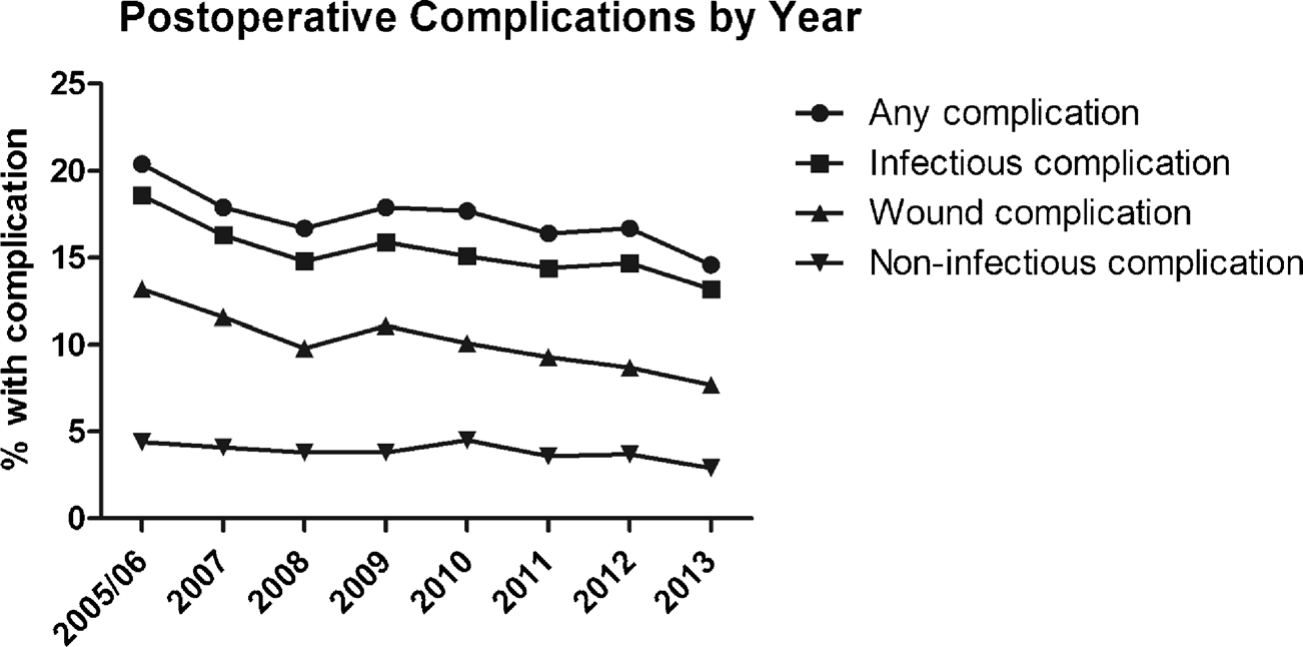
Trends in composite endpoints from 2005 to 2013

Figure 4 displays the results of the univariate analysis comparing rates of each individual postoperative complication across the eight yearly cohorts. The largest absolute reduction in postoperative morbidity was observed in relation to superficial SSIs, for which there has been a steady decline from 10.7 % in 2005/2006 to 6.0 % in 2013 (*p* < 0.001) (Table 4). Statistically significant downward trends over time were also identified for rates of deep SSI, pneumonia, reintubation, PE, failure to wean, renal failure, UTI, cardiac arrest, septic shock, and 30-day mortality (Table 4). The proportion of patients with LOS > 7 days has steadily declined from 24 % in 2005/2006 to 18 % in 2013 (*p* < 0.001), with a change in the mean LOS from 6.4 days (SD 5.9 days) to 5.6 days (SD 3.9 days). The only two complications that have increased in incidence over time were organ space infections (from 2.6 % in 2005/2006 to 3.6 % in 2013, *p* = 0.007) and renal insufficiency (0.3 to 0.5 %, *p* = 0.023) (Table 4). Similarly, analysis of the composite outcome endpoints demonstrated a statistically significant decline in infectious complications, non-infectious complications, wound complications, and any 30-day postoperative morbidity (Fig. 5).

**Table 4:** Complications by year in patients undergoing surgery for diverticulitis

Complication	2005/06, % (*n* = 1660)	2007, % (*n* = 2452)	2008, % (*n* = 2973)	2009, % (*n* = 3130)	2010, % (*n* = 3654)	2011, % (*n* = 4232)	2012, % (*n* = 5489)	2013, %(*n* = 6303)	*χ* ^2^ *p* value
Any complication	20.4	17.9	16.7	17.9	17.7	16.4	16.7	14.6	*<0.001*
Infectious complications	18.6	16.3	14.8	15.9	15.1	14.4	14.7	13.2	*<0.001*
Superficial SSI	10.7	9.5	8.0	9.3	8.1	7.4	7.0	6.0	*<0.001*
Deep SSI	1.6	1.5	1.2	1.2	1.2	1.3	0.9	1.1	0.341
Organ space SSI	2.6	2.7	2.3	2.7	3.2	3.4	3.5	3.6	*0.007*
Pneumonia	1.7	1.4	1.2	1.0	0.6	0.5	0.9	0.8	*<0.001*
Urinary tract infection	2.9	2.2	2.2	2.4	2.1	1.9	2.4	1.7	*0.037*
Sepsis	4.1	2.7	2.7	3.1	3.0	3.0	2.9	2.8	0.188
Septic shock	1.3	1.4	0.9	0.5	0.6	0.4	0.6	0.7	*<0.001*
Non-infectious complications	4.4	4.1	3.8	3.8	4.5	3.6	3.7	2.9	*0.003*
Dehiscence	1.3	1.2	0.9	1.2	1.3	0.9	1.1	0.9	0.369
Reintubation	1.4	1.1	1.0	0.6	0.8	0.8	0.6	0.6	*0.009*
Pulmonary embolism	0.6	0.4	0.4	0.5	0.5	0.4	0.4	0.3	0.411
Failure to wean	1.3	1.1	1.0	0.5	0.8	0.5	0.6	0.7	*0.007*
Renal insufficiency	0.3	0.2	0.3	0.2	0.7	0.5	0.5	0.5	*0.023*
Renal failure	0.4	0.4	0.4	0.3	0.2	0.3	0.2	0.2	0.619
Cerebrovascular accident	0.1	0.2	0.3	0.1	0.1	0.1	0.1	0.0	n/a
Cardiac arrest	0.2	0.2	0.1	0.2	0.2	0.2	0.1	0.1	n/a
Myocardial infarction	0.1	0.2	0.1	0.4	0.2	0.4	0.3	0.2	0.133
Deep venous thrombosis	0.6	0.9	0.7	0.6	0.8	0.7	0.7	0.5	0.274
Wound complications	13.2	11.6	9.8	11.1	10.1	9.3	8.7	7.7	*<0.001*
Mortality, 30-day	0.5	0.6	0.3	0.5	0.4	0.4	0.3	0.3	0.163
LOS >7 days	24.0	24.2	22.8	22.2	22.2	20.5	20.9	18.1	*<0.001*
Mean LOS, days (SD)	6.4 (4.1)	6.4 (4.3)	6.1 (4.1)	6.2 (4.3)	6.1 (4.4)	6.0 (4.2)	5.9 (4.2)	5.6 (3.9)	*<0.001* ^a^

Italics indicates *p*<0.05

*SSI* surgical site infection, *LOS* length of stay, *n/a* not applicable

^a^One-way ANOVA *p* value

**Fig. 6:**
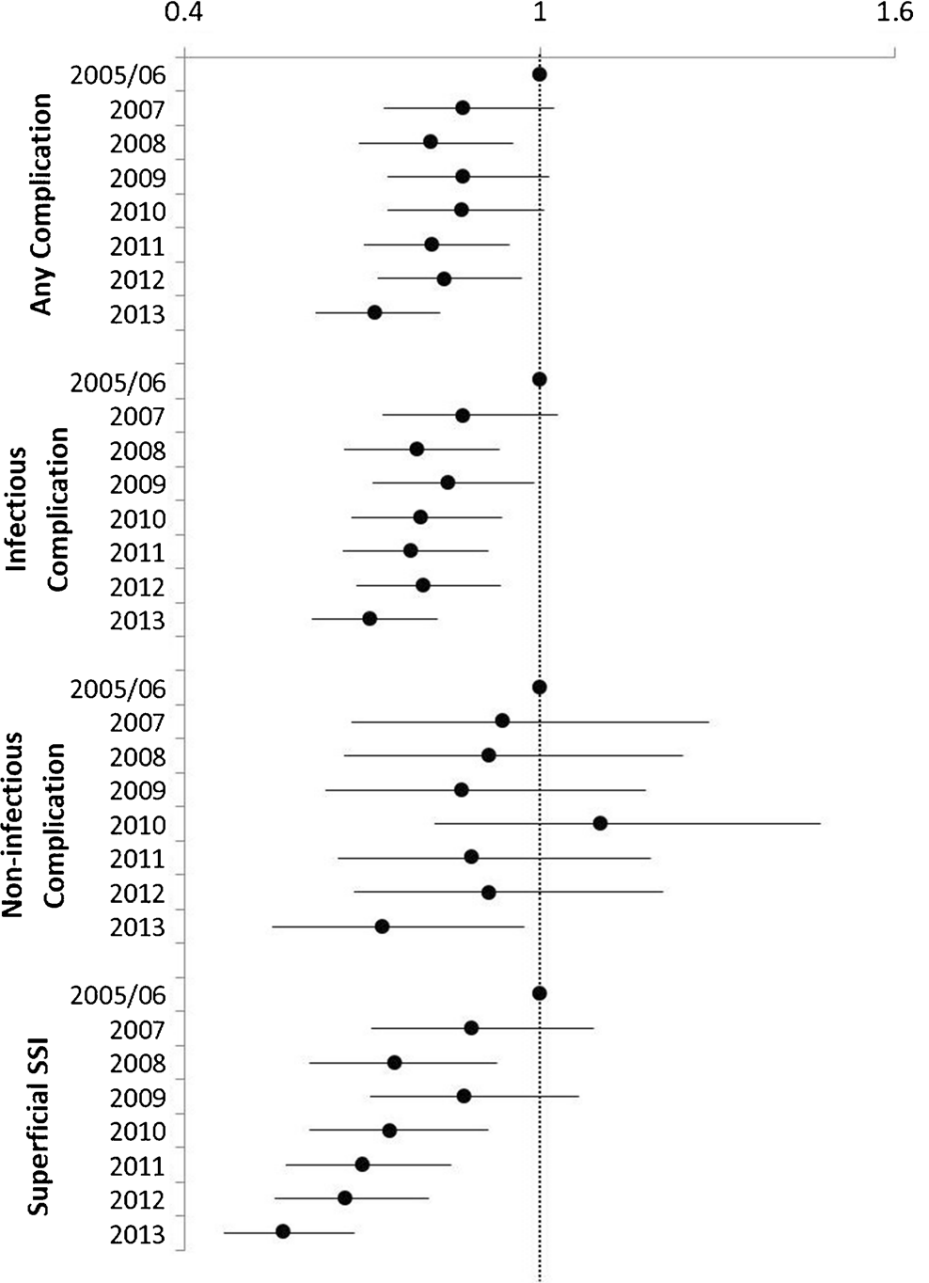
Results from multivariate analysis of outcomes controlling for gender, age, BMI, operative approach (laparoscopic vs open), diagnosis (diverticulitis vs diverticulosis), comorbidities, functional status, and ASA class. The 2005/2006 cohort is used as the reference group. Estimated odds ratios (indicated by the *solid circles*) and 95 % confidence intervals for the estimate are displayed

Multivariate analysis controlling for gender, age, BMI, operative approach, preoperative comorbidities, functional status, ASA class, and diagnosis further confirmed that operative year is an independent predictor of many of postoperative outcomes in this study (Fig. 6). In particular, the odds ratios for infectious complications have steadily declined down to 0.714 (95 % CI 0.615–0.828) for the 2013 cohort when compared to 2005/2006. Superficial SSI has decreased significantly with an approximately 50 % reduction in risk in 2013 compared to the reference (OR 0.567, 95 % CI 0.468–0.688). In the multivariate model, non-infectious complications have not significantly changed over time, except for the 2013 cohort, which had an OR of 0.731 (95 % CI 0.548–0.973) compared to 2005/2006.

**Fig. 7:**
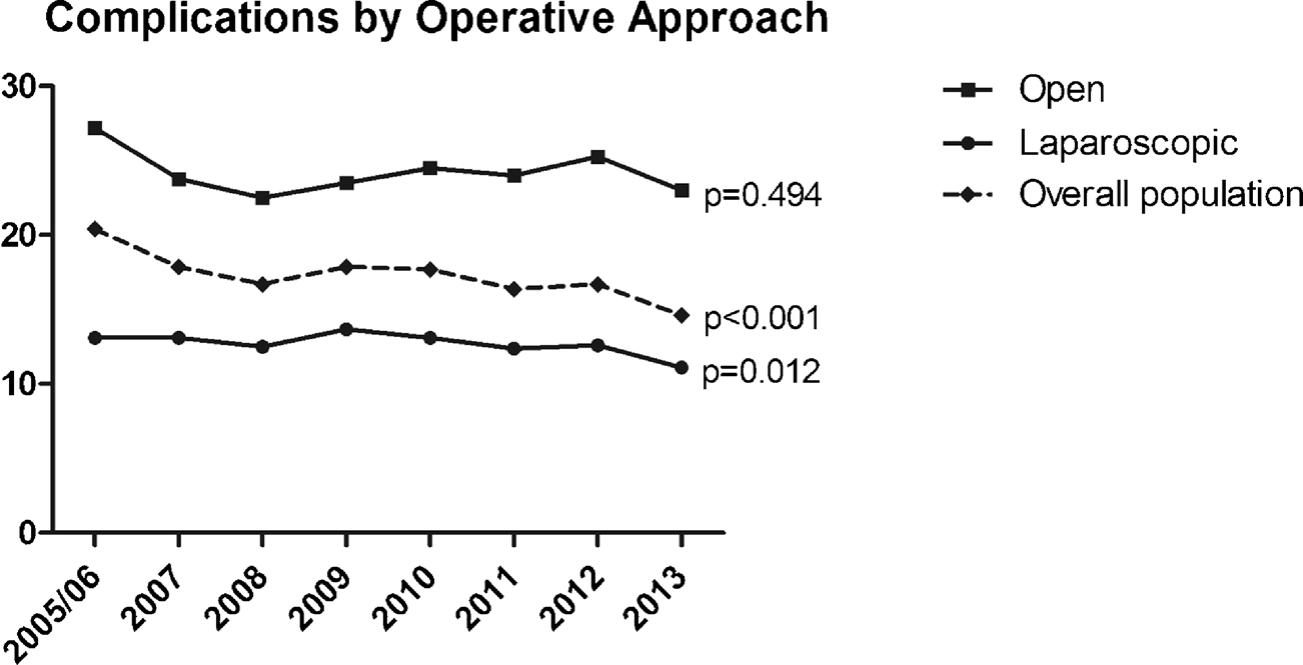
Univariate analysis showing rates of any postoperative complication across time, stratified by the operative approach. While the rates of complications within each category of operative approach have remained relatively stable over time, the rates of complications in the overall study population (*dashed line*) have decreased significantly. The *p* values are for Mantel-Haenszel tests of trend

Comparison of operative approach within each yearly cohort demonstrated significantly lower morbidity with laparoscopic compared to open surgery (Fig. 7). However, when stratified by the operative approach, there has been no change in rates of overall postoperative morbidity over time specifically among patients undergoing open surgery (chi-square, *p* = 0.227; Mantel-Haenszel, *p* = 0.494) (Fig. 7). There has similarly been very minimal reduction in complications among patients undergoing laparoscopic surgery (chi-square, *p* = 0.092; Mantel-Haenszel, *p* = 0.012) from 13.1 % in 2005/2006 to 11.1 % in 2013. Nonetheless, when the study population is examined as a whole, complications have significantly declined (dashed line, Fig. 7), suggesting that the increased adoption of minimally invasive techniques, rather than improved safety of the technique itself, has been a primary driver of improved outcomes.

## Discussion

Despite a general trend towards higher ASA class, older age, and higher BMI among patients undergoing non-emergent surgery for diverticular disease, there has been a trend towards lower 30-day morbidity and mortality in this patient population from 2005 to 2013. Notably, this decrease in postoperative morbidity is inversely related to the increasing use of laparoscopy.

The benefits of minimally invasive surgery for colon resection have been demonstrated repeatedly for many disease processes, including diverticulitis. Large retrospective cohort studies consistently report fewer postoperative complications, lower mortality, shorter hospital stays, and reduced cost when compared to open colectomy.^21^–^23^ Importantly, these findings have been confirmed in a randomized, controlled trial comparing laparoscopic and open approaches for sigmoid colectomy in the treatment of diverticulitis. The Sigma Trial, which included 104 patients undergoing elective sigmoid resection for diverticular disease, demonstrated a 15 % reduction in major complication rates, improved quality of life, and shorter hospital stay in patients randomized to a laparoscopic approach.^24^ Given the data that has emerged over the last decade demonstrating the superiority of a laparoscopic approach, it is encouraging to observe the steady increase in adoption of this technique within our study cohort.

The decrease in morbidity and mortality related to non-emergent surgery for diverticulitis over the last 10 years is certainly multifactorial. While the increased utilization of laparoscopy has clearly contributed to improved outcomes in patients undergoing colectomy for diverticulitis, this cannot fully account for the temporal trend in improved outcomes over time observed in this study. The multivariate analysis controlling for operative approach suggests that year of operation remains an independent significant predictor of short-term morbidity, even when controlling for operative approach. The explanation for this trend likely involves a complex interaction between changes in management practices surrounding diverticular disease as well as system changes and other quality improvement initiatives unrelated to patient or operative factors. Admittedly, a major limitation of this study is its inability to distinguish the contribution of each of these underlying factors over time. Nonetheless, this observation of improving outcomes over time is consistent with other large database studies. In a large, population-based analysis using the nationwide inpatient sample (NIS) database, Etzioni et al. found a reduction in surgical mortality and a decrease in hospital LOS over the study period from 1998 to 2005.^12^ In a similar analysis of NIS data from 2002 to 2007, Masoomi and colleagues reported a statistically significant decrease in both hospital LOS and mortality specifically among patients undergoing elective surgery.^13^ Finally, a recent study of Canadian administrative data reporting on outcomes following urgent surgery for diverticulitis from 2002 to 2012 similarly found an improvement in postoperative outcomes, including lower mortality and shorter hospital LOS.^5^ The rate of laparoscopy over this time period doubled from 9 to 18 %, which is consistent with the trend observed in our study, but at much lower rates compared to the increase from 48 to 70 % in our study, which may be due to different practice patterns in Canada or a biased sample that over-represents laparoscopy in the NSQIP database.

Interestingly, we found that rates of stoma creation have remained largely unchanged over time at around 10–12 % of patients undergoing non-emergent surgery. Prior studies have reported no change in the rates of colostomy formation in patients undergoing surgery in the acute setting.^5^,^12^ However, when stratified by the type of admission, authors have found a slight downward trend in the use of intestinal stomas in patients undergoing elective surgery.^12^,^13^ The rates of colostomy formation in these studies are also a little lower than those in the present cohort, ranging from 5 to 9.6 %. We did not differentiate between colostomy and diverting loop ileostomy, which likely accounts for the slightly higher rates of intestinal diversion observed here. The decision to perform intestinal diversion, whether in the setting of a Hartmann’s procedure or proximal diversion to protect a primary anastomosis, is complex and often based largely on factors at the time of operation, especially in the elective setting. It is possible that as more non-operative techniques are used in the acute setting in order to bridge patients to an elective operation, the population of patients over time undergoing so-called elective surgery actually have more complex disease, perhaps resulting in no observed difference in ostomy usage. The need for intestinal diversion, even in the elective setting, is unlikely to ever resolve completely.

While we have identified a clear trend in improving short-term postoperative outcomes from 2005 to 2013, our study has several limitations. First, in using the NSQIP database to analyze trends in postoperative outcomes, there is the possibility of confounding due to the very nature of the database itself. The main advantage of this database is the robust patient comorbidity and short-term outcome data allowing for both risk adjustment and more refined analysis of postoperative outcomes. However, this database is designed as a tool to improve the quality of surgical care delivered to patients by participating institutions, and therefore, it is difficult to ascertain the extent to which participation in this program alone contributed to our findings, rather than the evolving management of diverticulitis. On the one hand, there have been many examples of single institutions using NSQIP data to improve their outcomes,^25^ and participating hospitals appear to have improved surgical outcomes over time.^26^ However, there does not appear to be a significant difference in outcome trends between NSQIP participants and non-participants,^27^–^29^ which suggests that our findings may be representative of a broader trend in outcomes not merely specific to NSQIP participation.

Another limitation related to the use of the NSQIP database was our inability to differentiate between patients who underwent planned open surgery and conversion from laparoscopic to open surgery. Presumably, conversion typically occurs due to intraoperative complication or unanticipated difficulty during the case, which would bias a comparison of operative approaches. Nonetheless, our finding of improved outcomes in patients undergoing laparoscopic surgery is consistent with many prior reports. Furthermore, the primary goal of this study was to evaluate temporal trends in outcomes. Regardless of the inherent selection bias in operative approach, our findings suggest that the overall improvement in outcomes over time is at least partially related to the increased adoption of an operative approach that is associated with fewer complications.

The second major limitation of this study is its inability to identify the primary factors responsible for the observed trends in outcomes. While the univariate and multivariate analyses of operative approach provide compelling evidence for the role of laparoscopy in improving outcomes, there are certainly other major contributors that cannot be specifically identified in this study. For example, implementation of enhanced recovery pathways in colorectal populations would be expected to improve outcomes and certainly occurred during the period of this study between 2005 and 2013.^30^–^32^ The last decade has witnessed a growing body of evidence supporting the use of oral antibiotics prior to colon surgery, and this practice has been associated with significant reductions in wound infection rates.^33^–^36^ The gradual decrease in LOS over time may also have contributed to lower complication rates due to less exposure to hospital pathogens. Although the relationship between LOS and hospital-acquired infection is certainly reciprocal, shortening LOS could be a contributing factor for the trend in improved outcomes. Finally, it is also possible that the improvement in outcomes over time is not due to changes in practice patterns, but instead changes in the patient population. However, this seems like an unlikely explanation for several reasons. First, we found that between 2005 and 2013, the patients included in our sample became older, more obese, and with higher ASA scores, all of which would be expected to predict higher postoperative morbidity. Second, guidelines have increasingly encouraged a higher threshold for performing elective colectomy for diverticular disease, which would result in higher average complexity of those patients actually undergoing surgery. There does appear to be an increase over time in population-adjusted rates of both elective and emergency surgeries for diverticulitis in the USA.^37^ It is unclear, however, if this is due to an increasing burden of disease or failure of surgeons to follow evidence-based guidelines on operative indications.

Finally, we were limited in our ability to identify how disease complexity in this population has evolved over time. While the NSQIP database provides robust patient-level risk adjustment data, there is no information available on disease-specific severity. Ideally, this type of analysis would control for the number of prior episodes and severity of episodes (i.e., uncomplicated versus complicated); however, this was not possible in this study. Additionally, we were somewhat limited in our ability to discriminate between truly elective surgery and semi-elective/urgent surgery. Several exclusion criteria were used to select elective patients to the greatest extent possible, but nonetheless, the study population is likely more heterogeneous than a population of patients undergoing only true elective surgery. However, this likely did not affect the overall observations regarding trends in outcomes to a large extent.

Despite these limitations, we have found a clear trend in reduction in postoperative complications from 2005 to 2013 among patients undergoing non-emergency surgery for the diagnosis of diverticular disease. This improvement has occurred despite apparent increasing complexity of the patient population.

## Conclusions

This study demonstrates a significant reduction in postoperative complications from 2005 to 2013 among patients undergoing partial colectomy or colostomy non-emergently for diverticular disease. This improvement in outcomes has occurred in the setting of a dramatic increase in the adoption of laparoscopy during this same time period. The improved outcomes observed over time also suggest that patients are not being harmed by the use of more non-operative initial management and a higher threshold for proceeding to elective colectomy.
